# Investigating the Relationship between Fear of Failure and the Delivery of End-of-Life Care: A Questionnaire Study

**DOI:** 10.3390/nursrep13010014

**Published:** 2023-01-24

**Authors:** John S. Latham, Hannah Sawyer, Sarah Butchard, Stephen R. Mason, Kathryn Sartain

**Affiliations:** 1 Department of Clinical Psychology, Institute of Primary Care & Mental Health, University of Liverpool, Liverpool L69 3BX, UK; 2 Liverpool Hospitals NHS Foundation Trust, Liverpool L7 8YE, UK; 3 Department of Psychology, Institute of Population Health, University of Liverpool, Liverpool L69 3BX, UK; 4 Merseycare NHS Foundation Trust, Liverpool L34 1PJ, UK; 5 School of Medicine, Institute of Life Course & Medical Sciences, Faculty of Health & Life Sciences, University of Liverpool, Liverpool L69 3BX, UK; 6 York Hospitals NHS Foundation Trust, York YO31 8HE, UK

**Keywords:** physician, doctor, nurse, end of life, palliative, fear of failure, self-efficacy, psychology, medical education

## Abstract

Objective: To investigate whether fear of failure (FOF) influences a clinician’s perception of how confident and comfortable they are in their delivery of end-of-life (EOL) care. Methods: Cross-sectional questionnaire study with recruitment of physicians and nurses across two large NHS hospital trusts in the UK and national UK professional networks. A total of 104 physicians and 101 specialist nurses across 20 hospital specialities provided data that were analysed using a two-step hierarchical regression. Results: The study validated the PFAI measure for use in medical contexts. Number of EOL conversations, gender, and role were shown to impact confidence and comfortableness with EOL care. Four FOF subscales did show a significant relationship with perceived delivery of EOL care. Conclusion: Aspects of FOF can be shown to negatively impact the clinician experience of delivering EOL care. Clinical Implications: Further study should explore how FOF develops, populations that are more susceptible, sustaining factors, and its impact on clinical care. Techniques developed to manage FOF in other populations can now be investigated in a medical population.

## 1. Introduction

Care at the end-of-life (EOL), across all settings and clinical specialisms, is considered one of the most difficult aspects of medical practice [1]. Guidelines provide clinicians with recommendations for how to offer the most effective EOL care [2,3], and training in EOL care is now integrated into all undergraduate training for physicians [4] and nurses [5]. These guidelines and stipulations within the curricula from professional bodies reinforce the idea that good care at the EOL is the responsibility of all health care professionals and that prognostic disclosure should aim to be comprehensive, timely, and included regularly in conversations with the patient to ensure high-quality care [6].

Adhering to these recommendations has been shown to benefit both patients’ and clinical staff experiences of EOL processes and treatment. It helps to establish clear expectations, emphasises the importance of planning and preparing oneself and family, reducing travel and treatment costs for patients and medical professionals/services, and enables a more effective grief and bereavement process for family and medical teams alike [7,8,9]. Realistic discussions of prognosis, paired with early palliative care, have shown to decrease the overuse of unnecessary interventions and, perhaps counterintuitively, prolonged patient survival [10,11]. Improving EOL care is a pressing issue, given the acute increase in people predicted to die over the next decade and beyond [12].

### 1.1. Barriers to Effective EOL Care

Despite the effectiveness of these recommendations, there appear to be several barriers to implementing them in everyday practice [13,14]. Contrary to evidence-based guidance, medical teams may present patients with prognostic uncertainty under the guise of sparing them distress. For example, in a study of 1193 patients with incurable metastatic lung or colorectal cancer, 74% of patients believed that chemotherapy could cure their cancer due to obscure patient messaging, which led to a more traumatic experience at the EOL [15].

Several contextual factors have been presented to explain the barriers to effective EOL conversations, including the impact of cultural taboo [16], challenges with patient comprehension [17], or competing demands on clinician time and energy [18]. However, it appears that psychological factors may be more often central to this issue. These factors include healthcare staff heroism [19], a desire for paternal control [20,21], apathy as a form of abstention [22], fear of uncertainty [23], decision regret [24], a sense of expectation of oneself and others and the resulting fear of failure [25], death anxiety and the reminders of the mortality of self, friends, or family [18], and protecting oneself from the experience of grief [21]. All the above have all been linked with the avoidance of or difficulty with effective EOL communication in qualitative interviews with physicians in acute healthcare settings [26].

Of the identified psychological factors, to our knowledge, only one psychological construct has been empirically investigated in EOL settings. Death anxiety, a reminder of one’s own mortality and vulnerability to death, and the impact this may have on sharing in someone else’s EOL experience [27], has been shown to impact a clinician’s empathy, style of communication, level of detail within communication, and clinical decision-making [28,29,30]. However, the most recently published systematic review by Draper et al. (2019) investigating this construct was unable to find collective evidence to suggest that death anxiety is substantially responsible for limiting the application of taught skills, the implementation of guidance, and triggering avoidance of EOL conversations and care [13], indicating other factors may play a role in this phenomenon.

However, other psychological factors that have not yet been sufficiently studied in terms of EOL may be central to this issue, including fear and, more specifically, fear of failure (FOF). FOF was initially defined as one’s attempt to avoid feelings of shame or humiliation as a consequence of being unable to achieve a goal [31]. This definition was developed into a more cognitive-specific construct by Conroy (2001) [32], as a cognitive protective response to the anticipation of a threat to one’s ability or sense of worth, with the goal of avoiding any strong or difficult emotional reaction connected to the threat. FOF has extensively been shown to be a barrier to performance in settings outside of the clinical practice such as in education, business, and sport [33,34,35], but more research is needed to understand the barrier of FOF in clinical settings.

Feelings of failure may be elicited in clinicians when patients reject treatment, negotiations fail, or a patient’s body no longer responds to treatment. Research has shown that clinical staff who perceive a patient’s death as a personal defeat have been found to hold on to grief and avoid further experiences of death [36,37]. More specifically, qualitative research into FOF and medical professionals has revealed that clinicians may develop strategies that further enable avoidance, including avoiding certain specialisms that may induce threat or panic [17,38], even when this conflicts with their core values and hampers essential skill acquisition [19,39,40].

FOF has been successfully measured and evaluated across many populations (including business and sport) using the Performance Failure Appraisal Inventory (PFAI) [41], but the PFAI has yet to be used to understand the relationship medical populations might have with FOF. PFAI aims to measure how a person might feel and respond when faced with the threat of failure across five person–environment interaction dimensions. In contexts outside of medicine, high scores of FOF on the PFAI indicate high levels of FOF. This has been shown to influence the physical capacity to endure stress, the introduction of disadvantageous coping behaviours such as eating disorders and drug abuse, as well as increases in worry, depression, perfectionism, and antisocial behaviour [42]. Since the PFAI has been such a successful tool in identifying FOF in other populations, using it to identify FOF in medical professionals may provide important insight.

Given that EOL care can be characterised by effective communication and intervention, understanding this field may lead to greater experiences of care and wellbeing for patients in the last days and hours of life, and better outcomes for staff members involved in this care. Further, reducing stigma and the perception of failure may also generate a more open discussion about mistakes, thereby facilitating a more honest and open environment in healthcare and medical education settings.

### 1.2. The Current Study

This research study aimed to investigate the role of fear and anxiety in EOL care, namely whether FOF influences a clinician’s perception of their confidence and comfortableness with the delivery of EOL care. Furthermore, it also explores whether those who are more likely to encounter death in their workplace showed less FOF. It was recognised that other factors may affect EOL conversations, including role (doctor or nurse), gender, years of experience, and number of EOL conversations, and so these covariates were included in the analysis.

The hypothesis was that a higher total score on the PFAI (Fear of Failure; Predictor Variable) would relate to lower scores in confidence in EOL communication (Outcome Variable 1), confidence in EOL decision making (OV2), and confidence with involving others in decisions (OV3) and will show a relationship with higher scores for discomfort when working with people who are dying (OV4). A secondary hypothesis was that higher scores on each of the five PFAI subscales would also relate to lower scores on all outcome variables. 

## 2. Materials and Method

### 2.1. Study Sample and Setting

This study adopted a cross-sectional quantitative design approach, using a homogenous stratified self-selecting sample. The stratum subset was physicians (doctors) or nurses, who participated by completing a questionnaire distributed by email to all teams across two hospital sites and, as a COVID-19 adaption, to other physician and nurse online national hospital networks.

For our planned analysis, sample size was calculated using the software GPower [43] based on the effect sizes found in a study examining the relationship between perfectionism and FOF in athletes (f^2^ = between 0.27–0.47) [44]. However, it was decided that a more conservative effect size would be more appropriate in this study’s power analysis, as it was not clear whether the effect of FOF would be comparable between a population of athletes and the medical population being studied. Therefore, a medium effect size of f = 0.15 was utilised (alpha level = 0.05 and Power = 0.95, sample size = 89).

### 2.2. Recruitment

A link to an online anonymous questionnaire was distributed by email to team managers, who further distributed this to all their team members who met study inclusion criteria, across all specialties within two large hospital sites in the Northwest and Northeast of England. A COVID-19 pandemic adaption to the research was to include other local and national hospital networks known to the primary researcher. A Freedom of Information request was made to both the Northwest and Northeast hospital trusts to identify their hospital data on teams/specialisms working in areas with a high prevalence of patient death. This data guided further prompts by internal representatives in palliative, psychology, and research and development (R&D) hospital departments, to ensure the sample would be representative of those working with EOL patients. To encourage participation, GBP 5 vouchers for an online retailer were offered to participants, with funding for the vouchers provided by the Department of Clinical Psychology at the University of Liverpool.

Although the focus of many comparative studies of EOL treatment has been on physicians, due to their ultimate decision-making responsibility within care proceedings, specialist nurses (defined as nurses working at NHS UK pay grade Band 6—senior responsibility level—and above) were included in this study, as they offer a broader level of care, which may encompass emotional support as well as additional decision-making responsibility [45]. Table 1 shows the exclusion/inclusion criteria. The study was opened at the start of March 2021 and closed to recruitment in July 2021.

### 2.3. Ethical Considerations

Ethical approval was sought from the Doctorate in Clinical Psychology Research Committee, University of Liverpool, the sponsorship review panel, University of Liverpool (sponsor ref: UoL001588), NHS HRA review board (IRAS ref: 289310), and the R&D departments in each partner trust (Northwest ref: SP0564; Northeast ref: 289310). All methods were performed in accordance with the relevant guidelines and regulations. Consent from participants was recorded online and held in a secure database.

Information about participation and the nature of the research was accessible online, at the start of each questionnaire, and participants were required to confirm that they understood the objectives of the research, the inclusion and exclusion criteria, their rights as participants, the voluntary nature of the study, and the confidentiality of answers and data management before they were able to complete the study.

### 2.4. Procedure

Participants were sent the questionnaire link by email through distribution by managers of hospital teams. After pressing the link, participants were asked to read an information sheet outlining the objectives of the research, the inclusion and exclusion criteria, their rights as participants, the voluntary nature of the study, and the confidentiality of answers and data management. Following this, they were directed to a Qualtrics online questionnaire designed by the researcher. Participants were asked to provide their consent and anonymously complete demographic information (including NHS trust, gender, role, area of specialism, years of experience, a frequency guestimate of how many EOL conversations they had had per month) and complete three standardised self-report questionnaires.

### 2.5. Measures

The questionnaire contained three elements: the Self-Efficacy in Palliative Care Scale (SEPC; as adapted by Mason and Ellershaw, 2004) [46], the Thanatophobia Scale (TS) [47], and The Performance Failure Appraisal Inventory (PFAI) [41]. The PFAI was the predictor variable in the study analysis. The three subscales of SEPC were representative of outcome variables 1, 2, and 3. TS was representative of outcome variable 4.

### 2.6. Confidence with EOL Care

This was measured using the Self-Efficacy in Palliative Care scale [46], which assesses efficacy in three subscales: confidence in communication, confidence in decision making and patient management, and confidence in multi-professional teamworking. The SEPC has 23 items, measured on a Visual Analogue scale from ‘Very anxious’ to ‘Very competent’ over 100 units (0–100). Participants were asked to make a mark on a line between these two points to indicate their selection, with higher scores indicating more confidence in the construct being assessed. A mean score was calculated for each subscale. 

### 2.7. Comfortableness with EOL Care

This was measured using the Thanatophobia Scale [47], which assesses attitudes towards palliative care. The TS is a seven-point ordinal scale containing seven items, including response options of 1 (strongly disagree) to 7 (strongly agree), with higher scores indicating more discomfort with the construct being assessed. A total score was calculated for each participant.

The SEPC scale and TS have been shown to be valid and reliable assessment scales within clinical staff populations, with Cronbach’s alpha ranges of 0.84 to 0.85 and 0.92 to 0.95, respectively [46]. Within this study, the SEPC and TS questionnaires each began with the statement: ‘Presented are a series of statements that relate to issues and experiences that may be encountered when working with someone in the end stages of life. When answering the questions, we would like you to imagine how you think you would feel in relation to the issues and situations presented’.

### 2.8. Fear of Failure

The Performance Failure Appraisal Inventory (PFAI) [41] is a multidimensional measure of FOF developed from a meta-theory of emotions, examining FOF as a function of person–environment interaction, rather than a trait or state or global experience. The PFAI has been validated in many different languages and contexts and has been shown to assess a broad motivational disposition rather than context-specific motivation. It comprises 25 items that measure five dimensions (subscales) of threat appraisals associated with FOF: (1) fear of shame and embarrassment (FSE); (2) fear of devaluing one’s self-estimate (FDSE); (3) fear of having an uncertain future (FUF); (4) fear of important others losing interest (FIOLI); and (5) fear of upsetting important others (FUIO). The measure uses a five-point scale with response options of −2 (‘do not believe it at all’), 0 (‘believe it 50% of the time’), and +2 (‘believe it 100% of the time’). Within this study, all items were introduced with the phrase ‘In my medical practice…’. Subscale scores were calculated as instructed by the measure (total score across all items in the subscale divided by the number of items in the subscale), and then the total PFAI FOF score was all subscale scores added together.

### 2.9. Internal Consistency Reliability Analysis

**Figure 1:**
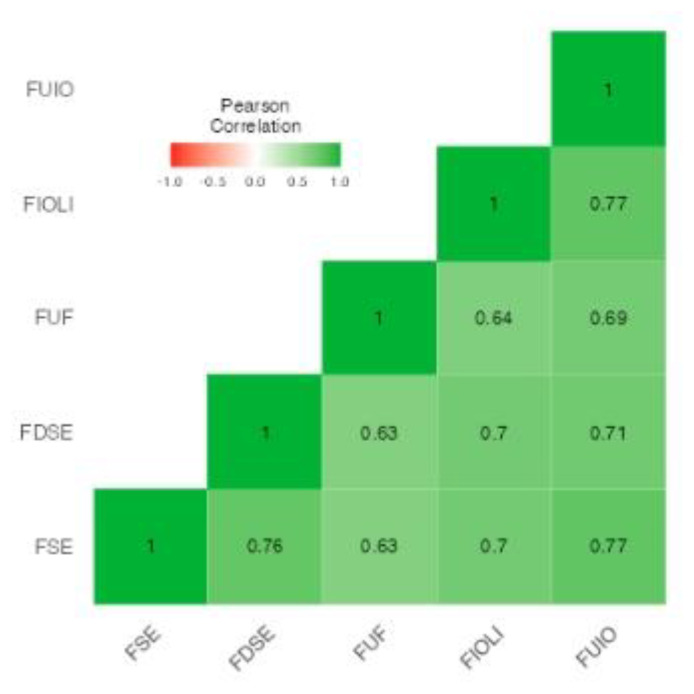
Correlation heatmap for 5 subscales of PFAI.

The PFAI consisted of 25 items across five subscales. All items were found to be highly consistent (α = 0.920; ω = 0.921), indicating that the subscales measured similar constructs and, overall, the PFAI is likely to be an effective measure when measuring FOF in medical populations. The strength of relationship between subscales of the PFAI is presented in Figure 1, which shows that there was a high correlation between all the subscales. Due to this, the individual subscales were tested against the outcome variables independently in the exploratory analysis.

### 2.10. Data Storage

Data were recorded, anonymised, and contained within the online Qualtrics platform. The anonymous data were then transferred for formatting using Microsoft Excel, held within a secure university filing system, and analysed using statistics software Jamovi version 2.3.18 (Internationally developed open-source project; Sydney, Australia), [48].

### 2.11. Planned Data Analysis

To test the hypothesis that the higher PFAI total score will relate to lower scores in terms of confidence in how participants perceive they communicate (OV1), make decisions (OV2), and work with others (OV3) and higher scores for discomfort (OV4), a hierarchical regression was conducted. Covariates (gender, role, area of specialism, years of experience, and a frequency guestimate of how many EOL conversations were had per month) were included in step 1. Step 2 then tested the potential relationships between FOF, as measured by the PFAI total score, and each outcome variable. The hierarchical regression was repeated, including an additional OV, total SEPC score, and with the total PFAI score replaced with the five subscales of the PFAI.

## 3. Results

### 3.1. Descriptive Statistics

A total of 205 questionnaire responses met the eligibility requirements and were included in the study. As can be seen from Table 2, there was almost an equal mix of physicians and nurses in the sample, and the majority of responses were from the Northwest NHS Trust. The total sample was skewed towards female respondents, which is reflective of the gender distribution in the healthcare workforce in the UK [49].

**Figure 2:**
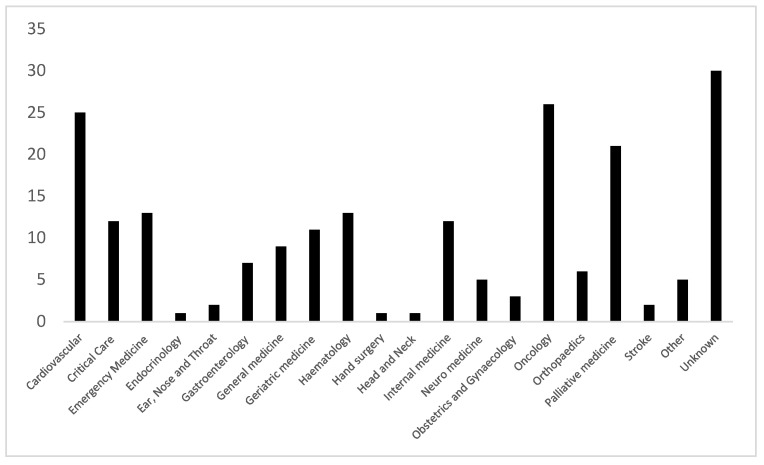
Distribution of clinical specialities of the sample.

Figure 2 shows the distribution of clinical specialities of the sample. As can be seen from the figure, the specialisms with the largest response rate included Oncology, Cardiovascular medicine, and Palliative medicine. Note that these groups were targeted during the recruitment process due to their high ward death rates; therefore, this distribution was expected. Geriatric medicine and Haematology were also targeted during recruitment due to death rates, but notably, they showed a lower response rate than other specialities. The unknown category describes participants who did not disclose their speciality. Further details can be found in Table A1, Appendix A.

Table 3 shows descriptive statistics for each of the outcome variables. As can be seen in the table, overall, the range of scores was generally large across all measures. 

Mann-Whitney U tests were conducted to see whether there was a significant difference between physicians and nurses and each of the outcome measures. There was no significant difference between the responses from physicians and nurses in terms of their reported scores on the PFAI (FOF) and Self-Efficacy measures. However, there was a significant difference on the Thanatophobia scale (*U* = 3579, *p* < 0.001), with physicians reporting higher levels of discomfort with death than nurses (see Table 3). This difference was found to be ‘medium’ as determined by the Cohen’s d effect size (Cohen’s *d* = 0.56). For further information, see Table A2 in Appendix A. 

### 3.2. Hierarchical Regressions

A hierarchical regression analysis was conducted to analyse the effect of participants’ self-perceived confidence in communicating with patients at the EOL (OV1) on perceived FOF (PV). To control for covariate variables (gender, role, years of experience, and number of EOL conversations), these were added in step 1. FOF was added in step 2. The overall regression model predicted approximately 8% of variance on perceived confidence in communication (adjusted R^2^ = 0.08, F(5,197) = 4.41, *p* < 0.001). The covariates predicted approximately 8% of variance in perceived confidence in communication, although only the number of EOL conversations per month was a significant predictor, indicating that those who had more conversations per month were less uncomfortable with EOL work. After controlling for the covariates, FOF did not account for any further variance. There was a similar pattern of results for OV2 (confidence in decision-making) and OV4 (comfortableness with EOL care). Although, for OV4, gender and role were also significant predictors, with male participants being less comfortable with death than female participants, and physicians were less comfortable than nurses. For OV3 (confidence in working with others), neither model was shown to be a good fit for the data. For further details, see Table A3 in Appendix A.

### 3.3. Exploratory Analysis

Since FOF did not account for any additional variance after controlling for covariates when a total PFAI score was used, the analysis then looked at whether the individual subscales predicted our outcome variables. Each subscale was analysed separately due to collinearity (Figure 1). The hierarchical regressions were performed such as in the main analysis, with the covariates (gender, role, years of experience, and number of EOL conversations) entered at step 1 and the subscale in question entered as step 2 (instead of total PFAI score). Overall, as shown in Table 4, this analysis demonstrated that at least one individual subscale of PFAI significantly predicted each of the outcome variables, even when a Bonferroni-corrected (0.05/5 = 0.01) alpha level was used. This suggests that, while the PFAI total score did not successfully predict the outcome variables, some subscales may still be helpful in determining specific aspects of FOF that clinicians perceive may be impacting the care they offer to EOL patients.

## 4. Discussion

This study identified that FOF, as a general concept and as defined by the PFAI, was not shown to have an impact on a physician’s or nurse’s perceived ability to deliver effective EOL care. Contrary to the hypothesis, after controlling for gender, role, years of experience, and number of EOL conversations per month, FOF did not impact confidence in communication, decision-making, team working, and comfortableness with EOL care. Estimated numbers of EOL conversations per month impacted three of the four outcome variables measured, and gender and role had an additional impact on how comfortable a clinician was with EOL care and death. The study also validated the use of the PFAI and its subscales within a new population group—medical professionals.

However, further exploratory analysis found that the subscales of the PFAI did show significant relationships with the outcome variables. For example, all OVs were negatively affected by fears of devaluing one’s self-estimate (FDSE) sub scale of the PFAI, which is a subscale that looks at self-depreciation, blaming lack of talent or intellect for a situation, and disappointment in self. The analysis also found that a fear of future uncertainty (FUF), or fear of a loss of interest in them and their professional opinion (FIOLI) negatively impacted confidence in communicating with patients, confidence in decision making, and comfortableness with EOL care. Furthermore, working with others was impacted by the FOF subscale Fear of Devaluing One’s Self-Estimate (FDSE), which is consistent with the literature suggesting that some professionals are reluctant to engage and work with palliative care teams in the care of their patients, due to the fear that this indicates the failure of their treatment [25,50].

Within this study sample, the overwhelming majority of EOL conversations were delivered by people with between 15 and 30 years of experience, and that age correlated positively with an increase in EOL conversations had with patients. Given that number of EOL conversations was shown to positively impact three of four OV’s, it may be suggestive that patient contact increases with age and that this increases confidence and comfortableness, or vice versa. This is in line with consumer feedback research that suggests that regular patient contact and engagement increases general and physiological knowledge, increases psychosocial content in patient interviews, reduces the fear of patient engagement and criticism over time and, for junior colleagues, improves medical exam performance [51].

Role and gender were both significant predictors of discomfort in perceived delivery of EOL care, with male physicians particularly impacted. Gender has often been linked in research with reluctance to talk about death, although it appears that little research exists that specifically explores clinical staff experience in acute hospitals. Men have been shown to be more likely to develop distress symptoms such as depression and grief anxiety if they fail to make meaning out of loss experience [52]. Men are also shown to be less expressive of grief and confide less in others compared to female counterparts [53]. It is possible that men and women are more similar than different in terms of many aspects of psychological processing [54] and, according to social role theory [55], they may simply have fewer opportunities for social support, in turn learning fewer ways to express emotion or are less familiar with being forthcoming about emotional experience and the benefits of vulnerability and social support.

Physician’s, more than nurses, were uncomfortable about working with a patient at EOL, although why this occurred is unclear, and it should be explored in further research. One possibility, however, is that medicine attracts people who see medicine as primarily a ‘problem solving’ profession, and traditional positivist approaches to medical education may validate this. If education structures dismiss the role emotions play in objective decision making, physician’s may be less likely to show an awareness of how events have affected them and their practice [56]. Further, if emotions are not considered a valuable form of knowledge, an emotional disconnect could occur, sanitising emotions into a set of behavioural skills, perhaps providing a greater illusion of objectivity [57]. Indeed, despite changes to the curriculum and teaching methods, graduates report feeling unprepared to provide EOL care, and anxiety continues to be a common theme [58].

### 4.1. Strengths and Limitations

In line with the literature, there may have been some reluctance from some specialities in completing the EOL questionnaire. Haematology has been previously identified as a speciality that ‘treats until the end’ and engagement with palliative teams can be seen as a sign of ‘giving up’ [17]. Despite the deliberate push in this study to recruit participants from these teams, including presenting at several team meetings, they were not as responsive as other targeted specialities. Other specialities that have very high death rates were also unexpectedly low in terms of response rate. Geriatric medicine and endocrinology are not often identified in the literature as specialities that experience reluctance; geriatric medicine reportedly works very closely with palliative care teams given the age of their population [16]. It may be that several factors influence response rates, including the COVID-19 pandemic, which put healthcare staff under unprecedented pressure, and caused the movement of physicians and nurses into specialties that were not familiar to them to meet the demand of the pandemic. A response rate comparison of participant area of specialism may also not account for specialities that have smaller teams, rather than a reluctance in these groups to engage. Unfortunately, data were not available to be able to include team size in the analysis. Participant culture and ethnicity were also not variables measured, which is an oversight of this study given the diversity within the UK healthcare workforce and the known impact culture has on one’s approach to death.

As described by Conroy (2001) [32], FOF can be viewed as a cognitive protective response to the anticipation of threat to one’s ability or sense of worth, with the goal of avoiding strong or difficult emotions. These same processes could have impacted the honest reporting of fears of failure, or may have contributed to cognitive dissonance, especially in relation to such a sensitive and often avoided topic. This could explain why the general FOF score was unable to produce a significant relationship with the SEPC and TS scales.

Increasing the number of participants and perhaps limiting the study to a single medical role may clarify any non-significant results. Observation studies running alongside self-reports could identify discrepancies in reporting and ensure that these are accounted for in subsequent analyses. In-depth interviews may help illuminate whether cognitive dissonance is indeed a limiting factor in questionnaire studies in this area.

### 4.2. Clinical Implications and Future Research

The unexpectedly positive response to this study may indicate the need for space to reflect about EOL processes and the psychological factors that impact them, perhaps created or propelled by the pressure on clinicians to support EOL patients during the COVID-19 pandemic. A development of this project would be to compare the responses captured in this study with a period in the future where the healthcare system is under less pressure in relation to dying patients and to reflect on the impact of acute periods of death on how EOL processes are perceived. Further, it may be interesting to identify whether differences can be observed between clinical specialties in terms of general FOF score or subscale scores. Given that there may be a cyclical effect where avoidance impedes confidence and comfortableness within this area, a robust distribution strategy is essential in targeting and recruiting specialties that are considered more reluctant to accept EOL care as a legitimate medical option. By comparing their response with other specialties, it may identify whether belief in the ability to cure shares similarities with avoidance to acknowledge the reality of death. Future research should also consider the role a healthcare staff member’s culture and ethnicity have on how death is experienced and whether this has an impact on the delivery of care to those dying. Patient culture, when different or similar to the healthcare worker, may also impact EOL care [16].

Whilst FOF is likely one psychological process affecting the delivery of EOL care, this study demonstrated that it does not explain all the variance in how confident and comfortable a clinician feels about delivering EOL care. Therefore, there remain unknown psychological factors affecting the delivery of EOL, and this offers a platform for the development of further research in this area. By understanding the psychological factors impacting EOL care, it may be possible to reduce their negative effects and consequently positively impact patient care.

There are several strategies that exist for managing fear and anxiety that have emerged from FOF studies in other settings, and the exploratory findings of this study indicate that it may be valuable to explore the use of these strategies within medical populations. Avoidance-focused strategies, such as forms of mindfulness, have been found to be a positive strategy when EOL rumination is knowingly affecting decision-making within the moment, but they rely on creating space for post-event processing [30]. Schwartz rounds are an example of spaces in health settings designed for sharing and reflection that have been found to be effective in reducing shame and acknowledging difficult health situations [59]. Emotion-focused coping strategies such as positive self-talk, positive reinterpretation, lowering goals, seeking emotional support, and problem focused strategies, such as increasing effort and education to prevent failure, and confronting salient fears have also been found to be effective strategies [2,23].

Further research could look at how to increase familiarity with EOL care for less confident staff, to explore strategies used by those who report less FOF, and to investigate effective fear-reducing strategies identified in other FOF literature from occupational psychology, sports psychology, and educational psychology.

## 5. Conclusions

Overall, this study has shown that FOF, as a general concept and as measured by the PFAI, is not representative of a single psychological factor impacting clinicians working in EOL care. Notwithstanding, four of the five subscales of the PFAI measure did represent some of the psychological factors that impact a clinician’s perceived delivery of EOL care. Despite significance, the models accounted for up to 18% of the data, indicating that other variables outside these factors are likely impacting the delivery of EOL care.

We demonstrated that psychological factors have an impact on both a physician’s and nurse’s confidence and comfortableness with EOL care. By understanding FOF in the context of EOL care, hospitals may be able to improve outcomes for patients. The scale and diversity of the sample, the validation of a new measure to capture clinician emotional experience, and the robust analysis using a two-step hierarchical regression have demonstrated links between aspects of FOF and the barriers clinicians face in confidence related to communication, decision-making, and comfortableness with EOL care. Given that FOF is an undeveloped psychological construct within this population, this research opens the opportunity for further exploration, including how aspects of FOF develop, sustaining factors, and other areas of clinical practice that FOF impacts. It also creates the opportunity to bring several research fields together, including research from business, education, and sports, which have already been developing the research base and exploring the impact of FOF on performance. Techniques that have been developed to offset the negative effects of FOF for other populations can now be researched and potentially applied to this medical population to see whether the same positive effects can be observed.

## Figures and Tables

**Table 1:** Inclusion/exclusion criteria for study.

Inclusion Criteria	Physicians and specialist nurses who have worked with adult EOL patients.
	Those who have access to a computer or smartphone.
	Location: The two specified north NHS trusts, or other local and national online networks.
Exclusion Criteria	Individuals who have not had an EOL conversation with a patient in their care.
	Clinicians working exclusively with children.
	Clinical staff who offer care support but who do not communicate information about EOL prognosis or who are not able to make autonomous decisions about patient treatment. Decision to include only Band 6 nurses (NHS scale, UK) or above as a result.

**Table 2:** Frequencies of participants by NHS trust, role, and gender.

NHS Trust			Role			Gender Distribution		
	Count	% of Total		Count	% of Total	Male	Female	Do Not Wish to Disclose
Northwest NHS Trust	86	41.9%	Physician	43	20.97%	31	12	0
			Nurse	43	20.97%	8	35	0
Northeast NHS Trust	58	28.4%	Physician	29	14.1%	17	11	1
			Nurse	29	14.1%	5	24	0
Other NHS Trust	61	29.9%	Physician	32	15.6%	15	17	0
			Nurse	29	14.1%	2	26	1
Overall Total	205		Physicians	104	50.7%	78 (38%)	125 (61 %)	2 (0.97%)
			Nurses	101	49.3%			

**Table 3:** Predictor variable and outcome variable mean scores, SDs, and ranges organised by role and gender.

Role	Gender	No.	PFAI Total—FOF Score (PV)			Perceived Confidence in Communication (OV1)			Perceived Confidence in Decision-Making (OV2)			Perceived Confidence in Working with Others (OV3)			Perceived Comfortableness with EOL Care (OV4)		
			Mean	SD	Range	Mean	SD	Range	Mean	SD	Range	Mean	SD	Range	Mean	SD	Range
Doctor	Combined	104	−0.42	0.70	3.26	60.55	24.16	92.33	63.48	20.87	86.14	62.72	22.83	92.57	23.29	9.76	39.00
	Male	63	−0.34	0.69	3.26	56.90	23.85	92.33	61.33	20.39	81.43	61.14	23.17	89.86	25.84	9.35	39.00
	Female	40	−0.59	0.71	2.93	66.47	24.05	86.25	67.11	21.59	83.00	65.74	22.33	82.86	19.38	9.28	34.00
	Did not disclose	1	−0.35	-	0.00	53.33	-	0.00	53.86	-	0.00	41.86	-	0.00	19.00	-	0.00
Nurse	Combined	101	−0.58	0.89	3.61	61.94	24.34	85.37	63.26	22.64	81.37	64.18	23.10	94.00	17.99	9.31	36.00
	Male	15	−0.49	0.71	2.48	65.30	25.79	78.50	61.94	23.53	71.29	61.67	23.13	72.85	19.33	10.12	27.00
	Female	85	−0.60	0.92	3.61	61.49	24.29	85.37	63.58	22.73	81.37	64.77	23.30	94.00	17.72	9.25	36.00
	Did not disclose	1	0.05	-	0.00	49.50	-	0.00	55.43	-	0.00	51.57	-	0.00	21.00	-	0.00

**Table 4:** Exploratory regression analysis showing gender, role, years of experience, number of EOL conversations, and the five subscales of the PFAI as predictors of each dependent variable.

Outcome Variable	Predictor Variable	Cumulative			Simultaneous		
		R^2^	Adjusted R^2^	*F*	β	SE	*p*
Perceived confidence in communication (OV1)	Step 1						
	Gender	0.10	0.08	F (4,200) = 5.63, *p* < 0.001	2.960	3.33	0.375
	Role				2.319	3.61	0.522
	Yrs. of experience				0.504	1.04	0.630
	No. of EOL conversations per month				4.718	1.09	<0.001
	Step 2 (Subscales of PFAI)						
	FSE	0.33	0.11	F (5,199) = 4.94, *p* < 0.001	2.329	1.63	0.155
	FDSE	0.17	0.15	F (5,199) = 7.97, *p* < 0.001	−7.175	1.81	<0.001
	FUF	0.14	0.12	F (5,199) = 6.61, *p* < 0.001	−6.295	2.04	0.002
	FIOLI	0.20	0.18	F (5,199) = 9.73, *p* < 0.001	−7.750	1.60	<0.001
	FUIO	0.15	0.13	F (5,199) = 7.23, *p* < 0.001	−6.004	1.71	<0.001
Perceived confidence in decision-making (OV2)	Step 1						
	Gender	0.11	0.09	F (4,200) = 5.98, *p* < 0.001	2.443	2.974	0.412
	Role				0.841	3.231	0.795
	Yrs. of experience				−0.834	0.933	0.372
	No. of EOL conversations per month				4.680	0.974	<0.001
	Step 2 (Subscales of PFAI)						
	FSE	0.11	0.09	F (5,199) = 5.06, *p* < 0.001	1.689	1.46	0.249
	FDSE	0.17	0.15	F (5,199) = 7.93, *p* < 0.001	−6.116	1.63	<0.001
	FUF	0.12	0.10	F (5,199) = 5.37, *p* < 0.001	−3.055	1.85	0.101
	FIOLI	0.13	0.11	F (5,199) = 6.17, *p* < 0.001	−3.729	1.49	0.013
	FUIO	0.12	0.10	F (5,199) = 5.58, *p* < 0.001	−2.978	1.56	0.058
Perceived confidence in working withothers (OV3)	Step 1						
	Gender	0.04	0.02	F (4,200) = 2.28, *p* = 0.062	1.438	3.25	0.659
	Role				2.259	3.53	0.523
	Yrs. of experience				−0.445	1.02	0.663
	No. of EOL conversations per month				3.120	1.06	0.004
	Step 2 (Subscales of PFAI)						
	FSE	0.04	0.02	F (5,199) = 2.06, *p* = 0.072	1.725	1.60	0.282
	FDSE	0.07	0.05	F (5,199) = 3.01, *p* = 0.012	−4.337	1.81	0.018
	FUF	0.05	0.02	F (5,199) = 2.03, *p* = 0.076	−2.068	2.03	0.310
	FIOLI	0.63	0.04	F (5,199) = 2.69, *p* = 0.022	−3.334	1.63	0.043
	FUIO	0.05	0.02	F (5,199) = 1.91, *p* = 0.094	−1.170	1.72	0.497
Perceivedcomfortableness with EOL care (OV4)	Step 1						
	Gender	0.11	0.11	F (4,200) = 7.33, *p* < 0.001	−3.763	1.338	0.005
	Role				−4.014	1.454	0.006
	Yrs. of experience				0.315	0.420	0.454
	No. of EOL conversations per month				−0.955	0.438	0.030
	Step 2 (Subscales of PFAI)						
	FSE	0.14	0.11	F (5,199) = 6.23, *p* < 0.001	−0.863	0.657	0.190
	FDSE	0.16	0.14	F (5,199) = 7.48, *p* < 0.001	1.991	0.744	0.008
	FUF	0.16	0.14	F (5,199) = 7.81, *p* < 0.001	2.408	0.822	0.004
	FIOLI	0.19	0.17	F (5,199) = 9.14, *p* < 0.001	2.491	0.656	<0.001
	FUIO	0.17	0.15	F (5,199) = 8.10, *p* < 0.001	2.169	0.691	0.002

Significant results are highlighted in grey within Table 4.

## Data Availability

All data analysed for this study are included in this published article as a supplementary information file. Data were deposited at the following Data Catalogue DOI: 10.17638/datacat.liverpool.ac.uk/1651.

## Appendix A

**Table A1:** Further details of the participant sample, showing clinical specialties, organised by trust and role.

		**Specialties/Departments**																			
		Cardiovascular	Critical Care	Emergency Medicine	Endocrinology	Ear, Nose and Throat	Gastroenterology	General Medicine	Geriatric Medicine	Haematology	Hand Surgery	Head and Neck	Internal Medicine	Neuro Medicine	Obstetrics and Gynaecology	Oncology	Orthopaedics	Palliative Medicine	Stroke	Other	Unknown
Northwest NHS Trust	Doctors	6	0	7	0	1	4	1	2	3	0	0	5	1	1	3	1	0	1	1	6
	Nurses	3	2	6	0	0	0	2	3	4	0	0	1	2	1	0	1	2	0	2	14
Total		9	2	13	0	1	4	3	5	7	0	0	6	3	2	3	2	2	1	3	20
Northeast NHS Trust	Doctors	7	0	0	0	1	1	3	2	2	0	0	3	1	0	0	2	2	1	0	4
	Nurses	3	0	0	0	0	1	2	1	1	1	1	2	1	0	6	0	8	0	1	1
Total		10	0	0	0	1	2	5	3	3	1	1	5	2	0	6	2	10	1	1	5
Other NHS Trust	Doctors	1	1	0	1	0	0	1	3	2	0	0	1	0	1	13	2	4	0	1	1
	Nurses	5	9	0	0	0	1	0	0	1	0	0	0	0	0	4	0	5	0	0	4
Total		6	10	0	1	0	1	1	3	3	0	0	1	0	1	17	2	9	0	1	5
	Total across Trusts	25 (12%)	12 (6%)	13 (6%)	1 (0.5%)	2 (1%)	7 (3%)	9 (4%)	11 (5%)	13 (6%)	1 (0.5%)	1 (0.5%)	12 (6%)	5 (2%)	3 (1%)	26 (13%)	6 (3%)	21 (10%)	2 (1%)	5 (2%)	30 (15%)

**Table A2:** Role as an effect on outcome variables as shown by Mann-Whitney and Cohen’s d.

					95% Confidence Interval	
	Statistic	df	*p*	Cohen’s d Effect Size	Lower	Upper
PV (PFAI-total FOF score)	4636	203	0.147	0.1657	−0.109	0.440
OV1 (SEPC-Communication)	5105	203	0.729	−0.0575	−0.331	0.217
OV2 (SEPC-Decision making)	5185	203	0.876	0.0104	−0.263	0.284
OV3 (SEPC-Working with others)	5096	203	0.714	−0.0633	−0.337	0.211
OV4 (TS-Discomfort working with death)	3579	203	<0.001	0.5552	0.270	0.838

**Table A3:** Regression analysis showing gender, role, years of experience, number of EOL conversations, and FOF, as predictors of the four outcome variables.

Outcome Variable	Predictor Variable		Cumulative		Simultaneous		
		R^2^	Adjusted R^2^	*F*	β	SE	*p*
Perceived confidence in communication (OV1)	Step 1						
	Gender	0.10	0.08	F (4,198) = 5.53, *p* < 0.001	3.585	3.83	0.350
	Role				2.072	3.77	0.583
	Yrs. of Experience				0.510	1.05	0.628
	No. EOL conversations per month				4.654	1.11	<0.001
	Step 2						
	Fear of Failure score (PFAI)	0.10	0.08	F (5,197) = 4.41, *p* < 0.001	0.399	2.07	0.848
Perceived confidence in decision-making (OV2)	Step 1						
	Gender	0.11	0.09	F (4,198) = 5.87, *p* < 0.001	3.025	3.42	0.378
	Role				0.523	3.37	0.877
	Yrs. of Experience				−0.845	0.94	0.369
	No. EOL conversations per month				4.625	0.99	<0.001
	Step 2						
	Fear of Failure score (PFAI)	0.11	0.09	F (5,197) = 4.77, *p* < 0.001	1.226	1.85	0.509
Perceived confidence in working with others (OV3)	Step 1						
	Gender	0.04	0.02	F (4,198) = 2.22, *p* = 0.068	3.233	3.73	0.388
	Role				1.268	3.68	0.731
	Yrs. of Experience				−0.481	1.02	0.639
	No. EOL conversations per month				2.951	1.08	0.007
	Step 2						
	Fear of Failure score (PFAI)	0.04	0.02	F (5,197) = 1.77, *p* = 0.120	0.227	2.02	0.155
Perceived comfortableness with EOL care (OV4)	Step 1						
	Gender	0.13	0.11	F (4,198) = 7.65, *p* < 0.001	−4.560	1.54	0.003
	Role				−3.663	1.51	0.016
	Yrs. of Experience				0.313	0.42	0.458
	No. EOL conversations per month				−0.876	0.45	0.050
	Step 2						
	Fear of Failure score (PFAI)	0.14	0.11	F (5,197) = 6.13, *p* < 0.001	0.347	0.83	0.677
